# Effect of the *PmARF6* Gene from Masson Pine (*Pinus massoniana*) on the Development of *Arabidopsis*

**DOI:** 10.3390/genes13030469

**Published:** 2022-03-07

**Authors:** Youju Ye, Xin Han, Hao Rong, Renjuan Qian, Jian Zheng, Zhouxian Ni, Li’an Xu

**Affiliations:** 1 Key Laboratory of Forestry Genetics & Biotechnology of Ministry of Education, Co-Innovation Center for Sustainable Forestry in Southern China, Nanjing Forestry University, Nanjing 210037, China; yeyj@njfu.edu.cn (Y.Y.); hanxin@njfu.edu.cn (X.H.); ronghao@njfu.edu.cn (H.R.); qrj@njfu.edu.cn (R.Q.); nzhx0627@njfu.edu.cn (Z.N.); 2 Wenzhou Key laboratory of Resource Plant Innovation and Utilization, Zhejiang Institute of Subtropical Crops, Wenzhou 325005, China; zjyzs@126.com

**Keywords:** *Pinus massoniana*, *Auxin Response Factor* (*ARF*), PmARF6, transgenic plant

## Abstract

Masson pine (*Pinus massoniana*) is a core industrial tree species that is used for afforestation in southern China. Previous studies have shown that *Auxin Response Factors* (*ARF*s) are involved in the growth and development of various species, but the function of *ARF*s in Masson pine is unclear. In this research, we cloned and identified Masson pine *ARF6* cDNA (PmARF6). The results showed that *PmARF6* encodes a protein of 681 amino acids that is highly expressed in female flowers. Subcellular analysis showed that the PmARF6 protein occurred predominantly in the nucleus and cytomembrane of Masson pine cells. Compared with wild-type (WT) *Arabidopsis*, transgenic *Arabidopsis* plants overexpressing *PmARF6* had fewer rosette leaves, and their flower development was slower. These results suggest that overexpression of *PmARF6* may inhibit the flower and leaf development of Masson pine and provide new insights into the underlying developmental mechanism.

## 1. Introduction

Masson pine (*Pinus massoniana*) is a core industrial tree species that is used for afforestation in southern China. This species has high overall utility and increasing economic benefits, and plays an irreplaceable role in the improvement of ecological environments. The wood fiber content of Masson pine is relatively high and is an important raw material for paper [1]. Pine resin is used in various industrial applications and typically consists of terpenoids [2,3]. In addition, the pollen of this species is used in medicine, cosmetics, and feed because it is hormone free and has a stable composition [4]. Therefore, the growth and development of Masson pine deserves further study. Transcription factor (TF) genes affect plant development by activating or inhibiting transcription. Some TFs of *P. massoniana* were predicted to be differentially expressed across different developmental stages in previous studies [5].

The core members involved in auxin signaling are *Auxin Response Factors* (*ARFs*), which are transcription factors (TFs) [6]. In general, *ARF*s consist of an amino-terminal DNA-binding domain (DBD), a middle region (MR), and a carboxy-terminal dimerization domain (CTD) [7]. It is worth noting that the MR contains either an activation domain (AD) or a repressor domain (RD), which could affect the function of *ARF* genes.

Previous reports have indicated that *ARFs* play core roles in the development of plants [8]. In the model plant species *Arabidopsis thaliana*, *ARF1* and *ARF2* regulate flower and leaf development [9]. In addition to its involvement in leaf development [10], *ARF3* is also closely related to the flower organ formation-related genes *AGAMOUS* (*AG*) and *APETALA2* (*AP2*). *ARF5* is involved in the formation of both root and flower organs [11]. *AtARF6* and *AtARF8* are redundant and regulate the development of flowers in *Arabidopsis* [12,13], and *ARF19* and *ARF7* are involved in leaf formation and lateral root development [14]. The functions of *ARF*s in other species have been identified. Studies of *ARF*s in banana show that *MaARFs* are involved in early fruit development [15]. In pineapple (*Ananas comosus*), *AcoARFs* participate in vegetative and reproductive organ development [16]. Similarly, *PeARFs* can regulate root development in poplar [17], and *TaARFs* (*Triticum aestivum*) likely participate in the disruption of flower growth [18]. Unfortunately, no *ARF*s have been reported in Masson pine.

In this research, we obtained the *ARF6* transcript from the transcriptome sequence of Masson pine and cloned its cDNA, analyzed its gene structure, and analyzed its spatiotemporal expression pattern in different tissues via real-time PCR. Transgenic lines were obtained through overexpression, and their regulatory roles in the flowering mechanism were analyzed. The results provide a basis for elucidating the regulatory mechanism underlying Masson pine development.

## 2. Materials and Methods

### 2.1. P. massoniana Plant Material

The female flowers, male flowers, roots, leaves, and stems of 15-year-old *P. massoniana* trees were collected from the *P. massoniana* clonal seed orchard established in Zhangping city, Fujian Province [19]. Each tissue was pooled together for RNA extraction. *Arabidopsis* (ecotype Columbia) plants were obtained by propagation in the laboratory. One-month-old seedlings of Masson pine were used as protoplast plant materials.

### 2.2. Total RNA Extraction and Coding DNA Sequence (CDS)

The total RNA of different tissues (1 mg) of *P. massoniana* was extracted with the RNeasy Plant Mini Kit (Tiangen, Beijing) [20]. The PrimeScript™ RT Master Mix Kit (Takara, Beijing) was used to synthesize cDNA. The full-length sequence was amplified using the SMARTer RACE 5′/3′ Kit (Clontech, Palo Alto, CA, USA) using the 5′ and 3′ primers reported in Table 1 and the following PCR conditions: 35 cycles of 98 °C for 10 s, 55 °C for 5 s, 72 °C for 10 s, and 72 °C for 3 min. The target fragment and open reading frame (ORF) was predicted by BioXM software and then verified by ORF-F and ORF-R primer. The PCR products were cloned using the Cloning Kit (Clontech, Palo Alto, CA, USA) and transformed into Trans1-T1 competent cells. Inserts from positive colonies were checked via PCR of the bacterial solution and sequenced [21]. Table 1 presents the primers used in this study.

### 2.3. Bioinformatic Analyses

The Gene Structure Display Server (http://gsds.cbi.pku.edu.cn/, accessed on 1 March 2022) was used to analyze the *PmARF6* transcript structure. Afterward, SOPMA software was used to predict the protein secondary structure. The NCBI (https://blast.ncbi.nlm.nih.gov/, accessed on 13 November 2021) was used to identify the similar sequences deposited in GenBank, and the Pfam databases (http://pfam.sanger.ac.uk/search, accessed on 12 November 2021) were used to identify the protein domains within a protein sequence. The DNAMAN 6.0 software was used to analyze the sequences. Finally, MEGA X software was used to construct the phylogenetic tree between the *PmARF6* and *ARF* genes in other species with the neighbor-joining method.

### 2.4. Protoplast Transfection in Masson Pine

Gateway technology (Invitrogen, Waltham, MA, USA) was used to construct the plasmids in this study [22]. The coding regions of the *PmARF6* gene without stop codons were cloned into the entry vector pCR8/GW/TOPO and the plant binary expression vector pBI121 (with a C-terminal HA-tag) driven by the cauliflower mosaic virus (CaMV) 35S promoter. Leaves of one-month-old *P. massoniana* seedlings were sliced into fine strips (0.5–1.0 mm). The strips were immediately transferred into freshly prepared enzyme solutions and incubated at 28 °C for 4–6 h in the dark. After enzymatic digestion, an equal volume of W5 solution (2 mM MES at pH 5.7, 154 mM NaCl, 125 mM CaCl_2_, 5 mM KCl) was added to stop the reaction. Protoplasts were released by filtering through 0.75 mm nylon meshes into round-bottom tubes using W5 solution. The pellets were collected by centrifugation at 100 *g* for 8 min. After washing once with W5 solution, the protoplasts were resuspended in MMG solution (4 mM MES at pH 5.7, 0.4 M mannitol, 15 mM MgCl_2_) at a concentration of 8 × 10^5^ cells mL^−1^. For each transient assay, 10 μg plasmid DNA was added to a 100 μL protoplast suspension, to which an equal volume of freshly prepared PEG solution was added (0.2 M mannitol, 100 mM CaCl_2_, and different concentrations of PEG 4000 (20, 30, and 40%); W/V). The mixture was incubated at room temperature for 30 min. After incubation, 1 mL of W5 solution was added slowly to stop the reaction. The protoplasts were collected by centrifugation at 150 *g* for 5 min. Then the protoplasts were resuspended gently in 100 μL of WI solution (4 mM MES at pH 5.7, 0.6 mM mannitol, 20 mM KCl) and then transferred to 24-well culture plates (Falcon) and incubated in the dark at 25 °C for 12–18 h. MLEG (http://mleg.cse.sc.edu/) was used to predict the subcellular localization of the PmARF6 protein. In addition, to observe the cellular localization of the fusion protein, confocal microscopy (Carl Zeiss, Oberkochen, Germany) with a fluorescence microscope excitation light source system (Lumen Dynamic Connections) was used. The protoplast transfection was repeated three times to ensure the accuracy of experiment.

### 2.5. Expression Analysis of the PmARF6 Gene by Real-Time PCR

The template consisted of diluted cDNA (threefold) of root, stem, and leaf tissue samples from *P. massoniana*. Oligo 7 was used to design qRT–PCR primers for *P. massoniana* (Table 1), and the experiment was then performed on an ABI Viia 7 system (Applied Biosystems, Waltham, MA, USA) in conjunction with a fluorochrome (Takara, Beijing, China) according to the manufacturers’ protocols [16]. Three technical replicates were performed for each sample. The 2^−ΔΔ^ CT method was used to calculate the relative expression levels in different tissues [23]. The *UXS* gene was used as a reference gene, and the specific primer sequence is shown in Table 1.

### 2.6. Transformation of Arabidopsis

The floral-dip method was used to transform the ORF of *PmARF6* into *Arabidopsis* (ecotype Columbia). Genetic transformation was mediated by *Agrobacterium tumefaciens* [24]. The coding regions of the *PmARF6* gene without stop codons were cloned into the entry vector pCR8/GW/TOPO and the plant binary expression vector pBI121 (with a C-terminal HA-tag) driven by the cauliflower mosaic virus (CaMV) 35S promoter, which is represented as 35S::*PmARF6*, including the HA-tag. The *PmARF6* plasmid was propagated for 18–24 h until OD600 = 1.8–2.0. Fresh 1/2 MS infection solution contained 5.0% sucrose and 0.05% Silwet L-77, pH = 5.7. The bacteria were collected and suspended in the infection solution. *Arabidopsis* inflorescence was resuspended in the infection solution for 30–45 s, and then left moist and away from light overnight before being moved to a normal growth incubator the next day. Transgenic lines were placed in a light incubator at 25 °C and 70% humidity with 16 h light and 8 h dark. After the T0-generation seeds were harvested, they were sown, and T1-generation strains were identified via PCR to obtain the positive plants for sowing and harvesting T2 generation. Additionally, PCR detection was used to obtain positive plants of T2 generation, and these seeds were sown to obtain the homozygous T3 generation. Then, we characterized the phenotype of T3 generation plants and detected the expression level during overexpression of the *PmARF6* gene. Genomic DNA was extracted from the leaves of *Arabidopsis* seedlings, and PCR was performed with specific primers that consisted of PmARF6 sequences (Table 1). The growth of wild-type (WT) and PmARF6-overexpressing *Arabidopsis* was observed at the same stage.

## 3. Results

### 3.1. Cloning and Analysis of PmARF6 in Masson Pine

**Figure 1:**
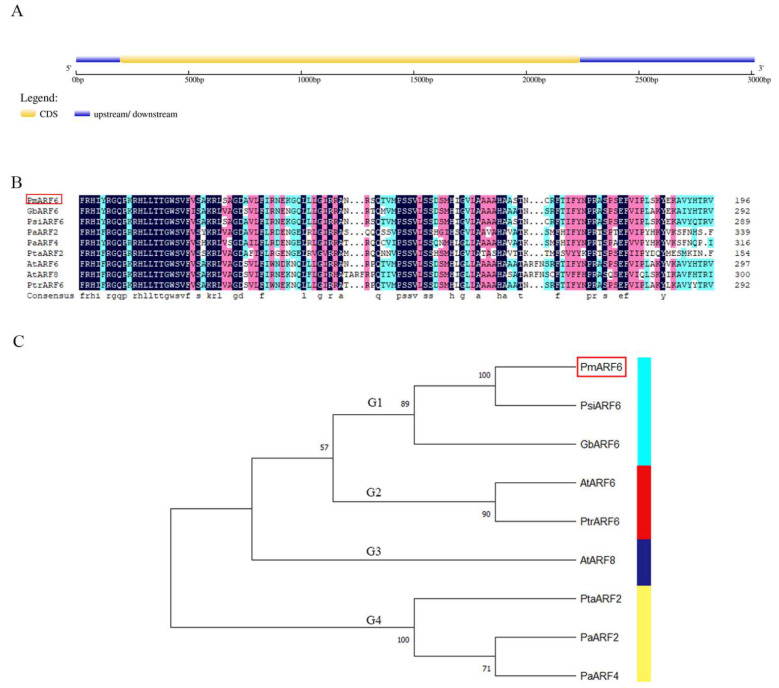
Sequence analysis of the *PmARF6* gene. (**A**) The gene structure of *PmARF6.* The 5′-UTR and 3′UTR are represented by the blue lines. The yellow line represents the 2043 bp ORF. Different cylinder-shaped colors represent different domains. The rectangle represents the Gln-rich region. (**B**) Sequence alignment of the ARF proteins. GenBank accession numbers: PsiARF6 (*Picea sitchensis* Psi012882), GbARF6 (*Ginkgo biloba*, CBA120031), AtARF6 (*Arabidopsis thaliana*, AT1G30330.1), PtrARF6 (*Populus trichocarpa*, Potri.001G358500.1), AtARF8 (*Arabidopsis thaliana*, AT5G37020.1), PtaARF2 (*Pinus taeda*, Pta011597), PaARF2 (*Picea abies*, MA_10432349g0010), and PaARF4 (*Picea abies*, MA_10431460g0020). (**C**) Phylogenetic tree of ARF6 proteins constructed with the neighbor-joining method in MEGA X. The G1, G2, G3 and G4 was represented by the light blue rectangle, red, dark blue and yellow, respectively.

Using RACE technology, we cloned *PmARF6*, which had a cDNA length of 3014 bp, a 195 bp 5′ upstream untranslated region (UTR), and a 776 bp 3′ downstream UTR (Figure 1A). The predicted TF *PmARF6* consisted of 681 amino acid residues. The molecular weight (MW) was 76.4 kDa, the isoelectric point (pI) was 7.57, and the grand average of hydropathy (GRAVY) value was −0.538; thus, the predicted protein was hydrophilic and acidic. The predicted protein secondary structure of *PmARF6* consisted of 29.96% α helices (Hh), 14.54% extended strands, 4.41% β turns, and 51.1% random coils, and the PmARF6 protein was mainly composed of random crimping and limbic helices. The Pfam database predicted that the PmARF6 protein domain contains a DBD at the amino terminus (N-terminus) and an MR with activation or inhibitory effects; the protein was not predicted to have a CTD (Figure 1A).

Sequence analysis indicated that *PmARF6* was highly similar to *AtARF6* and *AtARF8* (Figure S1). To observe the ARF protein relationships among *P. massoniana* and other plant species, a phylogenetic tree with 1000 bootstrap replications was constructed. The results show that the *ARF* family members from different plant species clustered into four groups (G1–G4). Within the ARF6 group, sequences from gymnosperms (PmARF6, PsiARF6, and GbARF6) formed a group (G1) distinct from the ARF6 of angiosperms (G2) (Figure 1C).

### 3.2. Subcellular Localization and Expression Levels of PmARF6 in Masson Pine

**Figure 2:**
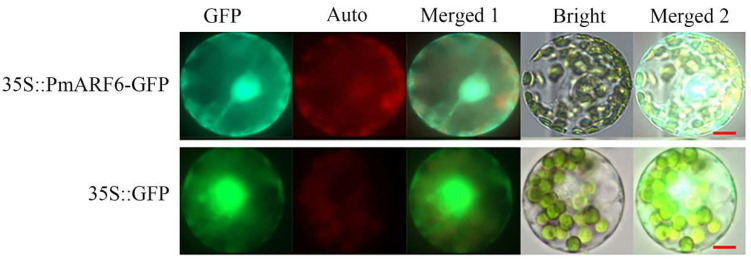
Subcellular localization analysis of the PmARF6 protein. The control protein is represented by the 35S-GFP fusion. Merged 1: GFP + Auto; Merged 2: Merged 1 + Bright; GFP: green fluorescent protein; Auto: chlorophyll autofluorescence; scale bar 10 μm, represented by the red line.

Subcellular localization is one of the most important component tools for validating protein function. MLEG predicted that PmARF6 is localized to the nucleus and dictyosome. To verify this prediction, we constructed 35S::GFP-*PmARF6* plasmids and inserted them into Masson pine protoplasts. Confocal microscopy revealed that the plasmids were located in the nucleus and cytomembrane (Figure 2). The 35S::GFP fusion protein was translated into the *P. massoniana* protoplast nucleus as a positive control.

**Figure 3:**
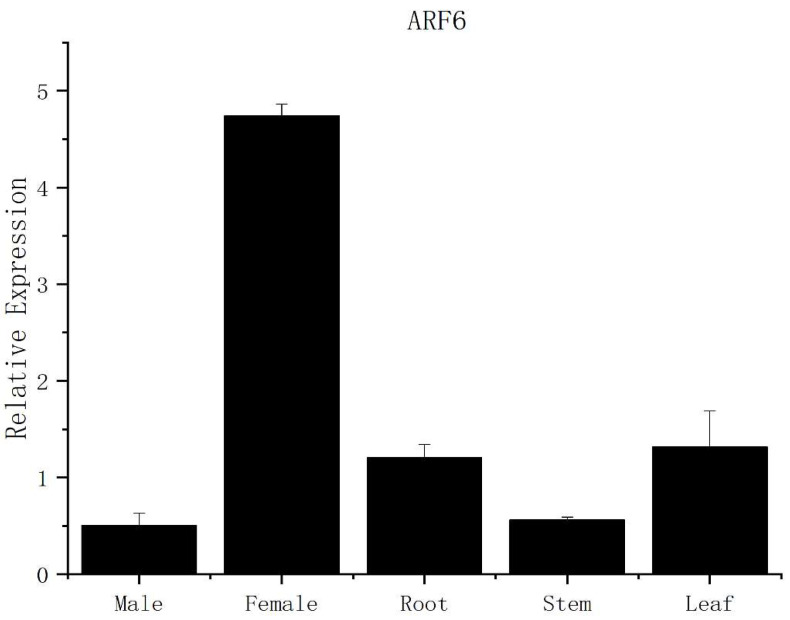
qRT-PCR results of the *PmARF6* gene. Male and Female represent male flowers and female flowers in *P. massoniana*, respectively. The error bars represent the standard deviations from three replicates.

Real-time PCR was used to measure the expression levels of the *PmARF6* gene in female flowers and male flowers, roots, leaves, and stems. We found that *PmARF6* expression was highest in female flowers, followed by leaves and roots. However, the expression in the stems and male flowers was approximately similar but was much lower than that in the female flowers (Figure 3).

### 3.3. Effects of PmARF6 Overexpression in Arabidopsis

**Figure 4:**
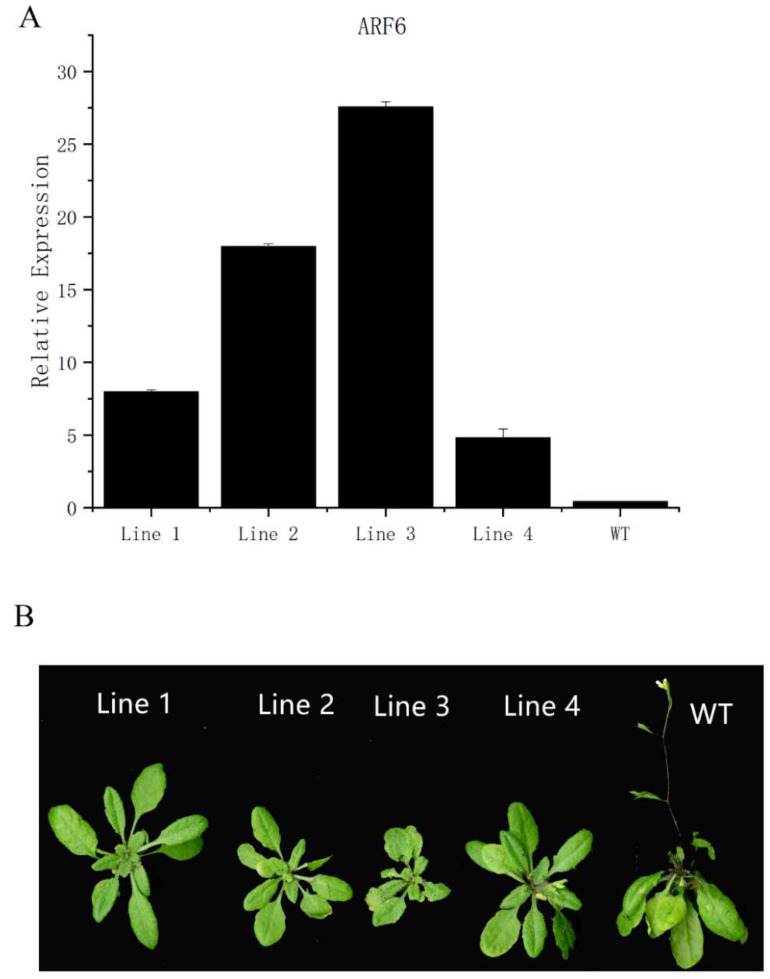
Expression level and growth status of 35S::*PmARF6* transgenic lines in *Arabidopsis*. (**A**) *PmARF6* gene expression levels in WT *Arabidopsis* (control) and PmARF6-overexpressing *Arabidopsis*. WT: wild-type *Arabidopsis*; L1–L4: lines 1–4 for transgenic *Arabidopsis*. The error bars represent the standard deviations of three replicates. (**B**) The growth phenotypes of WT *Arabidopsis* (control) and *PmARF6*-overexpressing *Arabidopsis*.

We identified the PCR amplicons and then measured the expression level of *PmARF6* in multiple 35S::*PmARF6* transgenic lines (L1–L4). The results indicate that the transgenic seedlings presented high expression levels of the *PmARF6* gene (Figure 4A). It is worth noting that the expression level of L3 was the highest, followed by that of L2.

Wild-type (WT) *Arabidopsis* and *PmARF6*-overexpressing *Arabidopsis* were subjected to long-day conditions to study whether the *PmARF6* gene affected growth and development. Through observation, we found that the *PmARF6*-overexpressing *Arabidopsis* plants grew slower than the wild-type plants. By 45 days after planting, the WT *Arabidopsis* had blossomed, whereas the transgenic line had no main stem. In addition, the transgenic lines had fewer leaves than the WT *Arabidopsis* (Figure 4B). The results of an additional comparative phenotypic analysis indicate that the number of rosette leaves for transgenic 35S::*PmARF6 Arabidopsis* L1 and L4 was similar to that for WT *Arabidopsis*; however, there were fewer rosette leaves for L2 and L3 than for WT *Arabidopsis*.

## 4. Discussion

*ARF* gene identification and analysis has been performed in many other plants, such as *Arabidopsis* [8], rice [25], and *Populus* [20], but studies on *ARF* genes in Masson pine are lacking. In this study, we cloned and identified the *PmARF6* gene at first. Then we analyzed its preliminary function by transgenic *Arabidopsis,* and the result showed that *PmARF6* gene effected the leaf and flower development in the *Arabidopsis.*

Sequence analysis indicated that *PmARF6*, which has two conserved domains (a DBD and an MR domain), showed high sequence similarity with AtARF6 proteins (Figure S1). Previous studies indicated that promoting the Aux/IAA protein might activate *ARF6* and that *AtARF6* regulates both stamen and gynoecium maturation [26]. The clustering of orthologous genes emphasizes that members within families may share the same functions [27].

The *PmARF6* expression level varied greatly across different organs but showed the highest expression level in female flowers. Previous studies indicated that *ARF6* is involved in the flower maturation process and that the *CmARF6* of *Cucumis melo* L. is highly expressed in female flower petals and may be involved in floral organ development [21,28]. Thus, the expression of *PmARF6* was the highest in female flowers, which may be due to *PmARF6* being mainly involved in female flower maturation. In addition, we conducted subcellular localization experiments of the PmARF6 protein in Masson pine, which has rarely been reported. We found that this protein was transiently expressed in the nucleus and cytomembrane.

In this study, to confirm that the *PmARF6* gene could affect flower and leaf development in transgenic plants, we induced high gene expression by infecting *Arabidopsis* flowers using *Agrobacterium* transformed with 35S::*PmARF6* constructs. Then, we evaluated four overexpression transgenic *Arabidopsis* lines (L1–L4). Among them, transgenic *Arabidopsis* L3 presented the highest expression level. Interestingly, the development of transgenic *Arabidopsis* L3 was the slowest among all the transgenic *Arabidopsis* lines. At present, the ARF family is one of the most widely studied TF families [29]. As an important member of the ARF family, *ARF6* may be involved in multiple processes of plant growth, such as fruit development, adventitious root development, flower bud growth, and leaf development [30]. In *Populus*, *PeARF6* participates in adventitious root development [16]. Moreover, in papaya, *CpARF6* plays a role during transcription in early cell division in flowers and fruit [31]. The L3 plant had the highest expression level, and its development was most notably affected. Notably, the expression level of *PmARF6* in L1 and L4 was low, and their developmental processes were also affected. This result indicates that the overexpression of *PmARF6* could affect flower and leaf development, which may lead to significantly delayed development in transgenic plants compared with the controls. Thus, the higher the expression of *PmARF6* is, the slower the plant will flower and produce leaves.

ARFs and Aux/IAA proteins interact to achieve their regulatory functions, as reported in previous studies. ARF proteins have a CTD located at the carboxy terminus (C-terminus); this CTD can bind Aux/IAA proteins. Domains III and IV of the Aux/IAA protein can form dimers by combining with ARFs [32,33]. The MR of ARFs is the AD or RD [34]. The RD is rich in a large number of leucine (L), serine (S), and glutamine (Q) residues, and the AD is rich in glycine (G), L, S, and proline (P) residues. According to these criteria, five *ARF* genes in *Arabidopsis* (*ARF5*/*ARF6*/*ARF7/ARF8/ARF19*) were considered to be transcriptional activators, and the others were considered transcriptional inhibitors [26]. In a report on *ARFs* in eucalyptus, 5 *EgrARFs* (*EgrARF5*, *EgrARF6A*, *EgrARF6B*, *EgrARF19A*, and *EgrARF19B*) were identified as transcriptional activators, and *EgrARF12* was identified as a transcriptional inhibitor [35]. We speculated that *PmARF6* is a transcriptional activator rich in G. Aux/IAA proteins that prevents ARF-mediated transcription by forming heterodimers with *ARF* activators in the absence of auxin [28,36]. However, *PmARF6* lacked a CTD region, which weakened the interaction with Aux/IAA in this research [37]. This hypothesis is relevant for *ARF* activators because *ARF*s have limited interactions with Aux/IAA proteins as repressors [36]. Thus, the insensitivity of *PmARF6* to auxin may lead to the delayed flower and leaf development observed in the transgenic plants compared with the WT. The specific underlying mechanism is thus worthy of future studies.

## Figures and Tables

**Table 1:** Primers for *PmARF6.*

Primer Name	Primer Sequence
5′ outer PmARF6	GAGATTGGGATGATGAGAGCTG
5′ inner PmARF6	GAATTCATAGAGAACCCAGA
3′ outer PmARF6	CGCCGTGCTGCTGAGAAAGTGT
3′ inner PmARF6	ATGACATTACAGCCGTTAAA
ORF-F	ATGACATTACAGCCGTTAAA
ORF-R	GAATTCATAGAGAACCCAGA
PmARF6-qF	GCGCTTCCGGATGCTGTTTG
PmARF6-qR	CTCGTGGCTGCCTCTCACC
*UXS*-qF	CGCCGGATTTCGCCCTCTTT
*UXS*-qR	CGTATCGGATCGGCCAAGGG

## Data Availability

The author confirms that the data supporting the findings of this study are available within the article and Supplementary File.

## Supplementary Materials

The following supporting information can be downloaded at: https://www.mdpi.com/article/10.3390/genes13030469/s1, Figure S1: Molecular phylogenetic tree of ARF6 protein between masson pine and Arabidopsis thaliana.
