# Supplementary material for: Effect of the PmARF6 Gene from Masson Pine (Pinus massoniana) on the Development of Arabidopsis
Source: Genes (Basel). 2022 Mar 7;13(3):469. doi: 10.3390/genes13030469 (PMC8949783; doi:10.3390/genes13030469)
Supplement: Supplementary file 1 [file genes-13-00469-s001.zip › genes-1580042-supplementary.pdf]

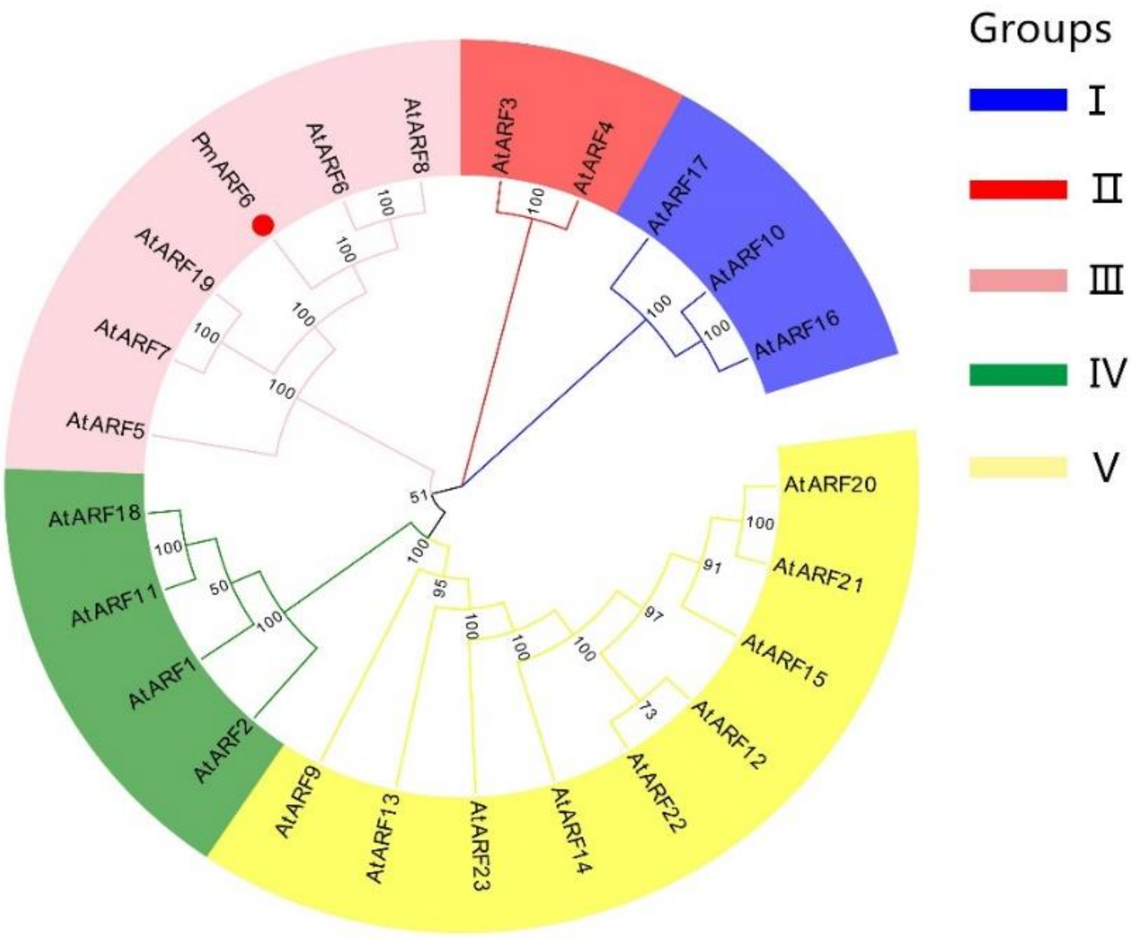

Figure S1. Molecular phylogenetic tree of ARF6 protein between masson pine and *Arabidopsis thaliana*.
